# Supplementary material for: Unlocking cinnamon export success: Key determinants from the world’s top five producers
Source: PLoS One. 2025 Dec 11;20(12):e0338601. doi: 10.1371/journal.pone.0338601 (PMC12697966; doi:10.1371/journal.pone.0338601)
Supplement: Attachment — Submitted filename: PONE_D_25_10270_Review Report.docx [file pone.0338601.s002.docx]

The manuscript, *"Key Determinants of Cinnamon Export Income: Insights from the World's Top Five Producers"*, explores the factors influencing cinnamon export income (CEI) for five major exporting countries (China, Sri Lanka, Indonesia, Madagascar, and Vietnam) over three decades. While the study provides valuable insights and has strong potential, it also has certain limitations that could be addressed to strengthen its contributions. Below is a critical review with suggestions for improvement and examples:

**Strengths**

1. **Timely and Relevant Topic**: The focus on cinnamon export income is pertinent, given the spice's global demand and economic significance. The emphasis on analyzing multiple countries adds depth to the investigation.
2. **Well-Defined Objectives**: The study clearly outlines primary and secondary objectives, aligning them with the research gap identified in existing literature.
3. **Data Coverage**: The analysis spans three decades (1992–2022), providing a robust time series for examining trends and relationships.
4. **Multifactor Analysis**: By incorporating production volume (PV), domestic consumption (DC), exchange rate (ER), and cultivated land area (CLA), the study provides a multidimensional view of the factors affecting CEI.

**Limitations and Suggestions for Improvement**

1. **Theoretical Framework**
   - **Limitation**: The theoretical framework connecting the identified factors to CEI is underdeveloped. While the study identifies PV, DC, ER, and CLA as key variables, it does not adequately explain the mechanisms through which these factors interact or influence CEI.
   - **Suggestion**: Introduce a conceptual model or flowchart showing causal relationships and interdependencies among the variables. For instance, explain how exchange rate volatility impacts both price competitiveness and market stability.
2. **Methodological Justification**
   - **Limitation**: The choice of a simple linear regression model may oversimplify the complex relationships among the variables. Factors like exchange rate and domestic consumption could have nonlinear impacts on CEI.
   - **Suggestion**: Consider adopting more advanced econometric models, such as panel data regression or structural equation modeling, to capture nuanced relationships and address potential multicollinearity.
   - **Example**: For exchange rates, explore whether their impact varies with changes in market conditions (e.g., fluctuations in global demand).
3. **Inconsistent Findings**
   - **Limitation**: The study reports conflicting impacts of PV and CLA on CEI across countries (e.g., PV has a negative impact in some countries but a positive impact in others). While these findings are interesting, the manuscript does not explore the underlying reasons for these differences.
   - **Suggestion**: Add a discussion on contextual factors (e.g., differences in trade policies, agricultural practices, or market structures) that could explain these variations.
   - **Example**: Investigate whether Indonesia's positive PV-CEI relationship is influenced by government subsidies or trade incentives.
4. **Limited Analysis of Policy Implications**
   - **Limitation**: The study briefly mentions implications for policymakers, but these are not detailed or actionable. For instance, recommendations for managing exchange rate volatility are lacking.
   - **Suggestion**: Provide concrete policy recommendations tailored to each country, focusing on areas such as foreign exchange risk management, technological innovation in agriculture, and export promotion strategies.
5. **Data Presentation**
   - **Limitation**: While the manuscript includes some descriptive statistics, the data presentation is limited in terms of visual appeal and explanatory detail.
   - **Suggestion**: Add more graphs, charts, or tables to visualize key findings. For example, use a comparative bar graph to show country-level differences in the impact of DC or CLA on CEI.
   - **Example**: A scatter plot illustrating the relationship between ER fluctuations and CEI across the study period would enhance reader comprehension.
6. **Narrow Scope of Study**
   - **Limitation**: The study focuses exclusively on cinnamon. While this adds specificity, the findings may not generalize to other spices or agricultural exports.
   - **Suggestion**: Acknowledge this limitation and propose future research extending the analysis to other high-value crops or spices to validate the generalizability of findings.
7. **Literature Review**
   - **Limitation**: The literature review does not adequately compare the current study's contributions to prior research, nor does it highlight its novelty.
   - **Suggestion**: Expand the literature review to include a critical assessment of gaps in prior studies and explicitly state how the current research fills these gaps.
8. **Language and Formatting**
   - **Limitation**: The manuscript contains instances of awkward phrasing and grammatical errors, which may detract from its professional presentation.
   - **Suggestion**: Perform thorough proofreading and editing to improve clarity and coherence.

**Examples of Specific Improvements**

- **Clarity in Data Interpretation**: Discuss why DC negatively impacts CEI in most countries but positively in Sri Lanka. Hypothesize that higher domestic consumption in Sri Lanka indicates robust internal demand, stabilizing export prices.
- **Improved Visualization**: Add a line graph showing annual CEI trends for each country, overlaying global cinnamon price indices for context.
- **Policy Insights**: Suggest that Sri Lanka adopt integrated marketing campaigns to leverage its unique position as a supplier of True Cinnamon.

By addressing these limitations and implementing the suggested improvements, the manuscript can significantly enhance its academic rigor, practical relevance, and overall impact.
