# Supplementary material for: Unlocking cinnamon export success: Key determinants from the world’s top five producers
Source: PLoS One. 2025 Dec 11;20(12):e0338601. doi: 10.1371/journal.pone.0338601 (PMC12697966; doi:10.1371/journal.pone.0338601)
Supplement: Attachment — Submitted filename: Author Response Document.docx [file pone.0338601.s003.docx]

**Reviewer #1 comments – Response Sheet**

| **Reviewer comments** | **Author response to reviewer’s comments** |
| --- | --- |
| **Theoretical Framework**   - **Limitation**: The theoretical framework connecting the identified factors to CEI is underdeveloped. While the study identifies PV, DC, ER, and CLA as key variables, it does not adequately explain the mechanisms through which these factors interact or influence CEI. - **Suggestion**: Introduce a conceptual model or flowchart showing causal relationships and interdependencies among the variables. For instance, explain how exchange rate volatility impacts both price competitiveness and market stability. | According to your comment, we have added the conceptual framework and justified the causal relationships and interdependencies (direct and indirect) among those variables as follows.  This is the conceptual framework added to the revise manuscript. **(Line 297 in the revised manuscript)**  Cinnamon production  Domestic consumption  Exchange rate  Harvested land area  Cinnamon export income  With the figure above, the following justification has been provided.  **(Line 299-316 in the revised manuscript)**  *“Accordingly, it is noted that, cinnamon export income (CEI) is affected by an interplay of different factors including price dynamics, macroeconomic variables, and production factors. Exchange rate (ER) fluctuations play a dual role: ER depreciation tends to bring exports more competitive internationally, boosting demand, a pattern confirmed in empirical studies on Indonesian spice exports [102]. However, exchange rate volatility introduces uncertainty in revenue expectations, which discourages long-term contracts and deters participation in volatile markets a pattern consistent across agri-export economies [101].*  *In addition, production volume fluctuations (PV) of spices such as cinnamon significantly increase trade risk by altering the dependability of export supply, increasing uncertainty of producers' revenues, and undermining the determinacy that is involved in participating in market transactions [103].On the supply side, domestic consumption (DC) is a key driver of the volume of crops available for export, as a rise in internal demand usually diverts production from global markets [99]. Finally, the size of cultivated land (CLA) is an indicator of the level of production expected for cinnamon. Although an expansion in land use reflects higher export potentials, its impact is subject to productivity rates, compliance with export regulations, and market orientation [100]”* |
| **Methodological Justification**   - **Limitation**: The choice of a simple linear regression model may oversimplify the complex relationships among the variables. Factors like exchange rate and domestic consumption could have nonlinear impacts on CEI. - **Suggestion**: Consider adopting more advanced econometric models, such as panel data regression or structural equation modeling, to capture nuanced relationships and address potential multicollinearity. - **Example**: For exchange rates, explore whether their impact varies with changes in market conditions (e.g., fluctuations in global demand). | We appreciate the reviewer’s insightful comment regarding methodological refinement. We acknowledge that simple linear regression may not fully capture the dynamic and potentially nonlinear relationships between the independent variables and CEI.  The choice of SLR was made intentionally for the following reasons:   - The study aims to present a country-wise comparative analysis using disaggregated time-series data (1992–2022), where panel estimation was constrained due to data unavailability or inconsistencies across countries and years. - SLR enabled us to isolate and visualise the country-specific impacts of each factor, offering an accessible and interpretable first-stage analysis.   That said, we agree with the concern and have accordingly:   - Included a clearer justification for our model choice under the "Methodology" section.   **(Line 360-367 in the revised manuscript)**  *“While the study employs SLR models for each country to estimate the relationship between CEI and its potential determinants (PV, DC, CLA, ER), this approach was selected to ensure interpretability and manage country-specific heterogeneity. However, we acknowledge that SLR may not capture possible nonlinearities, interactions, or cross-country dynamics. Due to limitations in data uniformity across the full sample, advanced techniques such as panel regression or structural equation modeling were deemed infeasible within this study’s scope. These limitations are discussed under the recommendations for future research.”*   - Acknowledged the limitation of SLR in the “Limitations and Future Research” section.   **(Line 819-828 in the revised manuscript)**  “*While this study provides valuable insights into the determinants of cinnamon export income across five major producing countries, several methodological limitations must be acknowledged. The use of SLR was chosen for its interpretability and due to constraints in consistent time-series data across countries; however, this approach may not fully capture the complex, potentially nonlinear relationships between variables or account for non-stationarity. As the dataset spans three decades, issues such as spurious regression arising from non-stationary data cannot be ruled out. Future research could address this limitation by employing more advanced econometric techniques such as panel data regression models, nonlinear specifications, or structural equation modeling to better understand dynamic interactions and control for country-specific effects.”*   - Outlined the scope for future research using panel data models, nonlinear estimations, or interaction terms (e.g., ER × Global Demand Index) to capture such nuanced effects.   **(Line 829-835 in the revised manuscript)**  *“Additionally, models such as the Autoregressive Distributed Lag or Error Correction Model can help account for unit roots and distinguish between short- and long-run relationships. Incorporating interaction terms (e.g., exchange rate × global demand shocks) and testing for multicollinearity using variance inflation factors would also enhance the robustness of future analyses. Addressing these methodological concerns would improve statistical validity and offer deeper insights into the causal mechanisms driving agricultural export performance.”*  We also examined the data for multicollinearity (using correlation matrices, not reported due to space) and observed moderate correlation values, which we acknowledge require deeper exploration in a multivariate framework.  We hope this clarification satisfies the reviewer’s concern and improves the transparency of our methodological choices. |
| **Inconsistent Findings**   - **Limitation**: The study reports conflicting impacts of PV and CLA on CEI across countries (e.g., PV has a negative impact in some countries but a positive impact in others). While these findings are interesting, the manuscript does not explore the underlying reasons for these differences. - **Suggestion**: Add a discussion on contextual factors (e.g., differences in trade policies, agricultural practices, or market structures) that could explain these variations. - **Example**: Investigate whether Indonesia's positive PV-CEI relationship is influenced by government subsidies or trade incentives. | Thank you for your comment and we have included paragraphs to justify the variances identified through results.  **(Line 727-745 in the revised manuscript)**  *“The heterogeneity in how PV, DC, ER CLA, and CEI interact across top cinnamon-producing countries reflects divergent structural, policy, and market contexts. In Vietnam, PV negatively impacts CEI primarily because output increases during global price volatility yield diminishing export revenues. This is explained by exchange rate volatility, which long-term suppresses exports. In addition, [105] show a 1% rise in ER volatility reduces export volume by approximately 0.11% and by expanding DC that diverts cinnamon from export channels. In China, similar dynamics apply rising domestic demand and currency strength can offset the gains from higher PV, leading to diminished CEI. Contrastingly, Indonesia leverages PV and ER positively because depreciation enhances competitiveness, and its production increases translate effectively into CEI due to strong export linkages and government support [99], [104]. In Sri Lanka, smallholder systems mean PV often undermines CEI, as increased CLA yields may not overcome price shocks and high DC, and occasional ER overvaluation may further erode export profitability [107]. Meanwhile, in Madagascar, PV and ER boost CEI when integrated with export-oriented systems, and CLA expansion efficiently scales exports due to available land and agro-ecological suitability [106]. These divergent findings underscore those variables like PV, ER, and CLA do not act uniformly; their net impacts depend on each nation’s trade policy, market structure, and scale capacities”* |
| **Limited Analysis of Policy Implications**   - **Limitation**: The study briefly mentions implications for policymakers, but these are not detailed or actionable. For instance, recommendations for managing exchange rate volatility are lacking. - **Suggestion**: Provide concrete policy recommendations tailored to each country, focusing on areas such as foreign exchange risk management, technological innovation in agriculture, and export promotion strategies. | As per the suggestion we have revised the policy implications for policymakers focusing country specific recommendations.  **(Line 761-777 in the revised manuscript)**  *“To enhance CEI, policy responses must be tailored to the unique interactions of PV, DC, ER and CLA across countries. In Vietnam, where PV and ER negatively affect CEI and rising DC reduces export availability, policymakers should prioritize exchange rate hedging tools, input subsidies, and minimum export quotas. China faces similar PV and DC-related constraints; thus, optimizing export infrastructure and balancing domestic–export demands through dynamic allocation systems could improve outcomes. Indonesia’s positive ER and PV impacts on CEI suggest maintaining currency-aligned export incentives, while reversing the adverse CLA effect may require transitioning from land expansion to yield intensification via smart agriculture. In Sri Lanka, the negative influence of PV and CLA underscores the need for price stabilization mechanisms and land productivity investments, whereas leveraging positive ER and DC impacts calls for deeper domestic value chain integration. Madagascar, benefiting from positive PV, ER, and CLA effects, should focus on export logistics, sustainable land expansion, and domestic consumption monitoring. These targeted strategies highlight that improving CEI requires context-specific reforms in land use, market access, currency risk management, and production planning.”* |
| **Data Presentation**   - **Limitation**: While the manuscript includes some descriptive statistics, the data presentation is limited in terms of visual appeal and explanatory detail. - **Suggestion**: Add more graphs, charts, or tables to visualize key findings. For example, use a comparative bar graph to show country-level differences in the impact of DC or CLA on CEI. - **Example**: A scatter plot illustrating the relationship between ER fluctuations and CEI across the study period would enhance reader comprehension. | Thank you for your valuable comments and seeing the possibility of further illustrations can be done using data in the current study, authors have done a correlation analysis before the linear regression models to see the connectedness among these factors considered impacting in CEI.  Accordingly, the following figure and the content included in the section “Results and Discussion”  **(Line 575-611 in the revised manuscript)**  “***Comparative analysis of key cinnamon export drivers by country***  *A deep understanding of the specific roles that key variables play in different national contexts is a must to identify the determinants of the success of cinnamon exports. To outline the unique strategies each country adopts to maximize their assets, here it has checked the correlations specific to each country between CEI and ER, CLA, PV and DC.*  ***Figure 12 : Country level correlation analysis***  *The analysis identified that Sri Lanka as the leading exporter, highlighting especially cinnamon, and displays a strong positive relationship between the CEI and both the CLA and DC. This finding highlights Sri Lanka's significant agricultural and economic commitment to the cultivation of cinnamon, where increased land allocation and increased DC are linked to increased export revenues. Vietnam presents a moderate positive relationship between CLA and CEI, representing some alignment between land utilization and returns on exports, but not as strong as the relationship evident with Sri Lanka. Likewise, Madagascar shows a high relationship between DC and CEI, illustrating domestic consumption and production patterns playing an influential part within export activity. China and Indonesia, with their larger GDP and variable exchange rates, display no strong connection with CEI, indicating the economic significance of cinnamon is limited within both countries. Overall, nations with increased land allocation and internal resources towards cinnamon cultivation (Sri Lanka, with some contribution from Madagascar and Vietnam) have demonstrated higher relative export revenues, highlighting the significance of committed agricultural endeavour over economics as scale as a determining factor within cinnamon export.”* |
| **Narrow Scope of Study**   - **Limitation**: The study focuses exclusively on cinnamon. While this adds specificity, the findings may not generalize to other spices or agricultural exports. - **Suggestion**: Acknowledge this limitation and propose future research extending the analysis to other high-value crops or spices to validate the generalizability of findings. | Much appreciating your comments and we have amended the limitations of the study referring narrowness of the study considering only cinnamon. We have added the following part to the existing content.  **(Line 809-818 in the revised manuscript)**  *“Though this research makes important contributions to knowledge of the determinants of cinnamon export earnings for the top five producers worldwide; however, its narrowness of focus is deliberate as it is limited to a single high-value spice. This specificity increases contextual richness but also narrows the externality of the empirical findings to other agricultural exports. Consequently, future research would need to be done with an expanded range of export-based spices as well as horticultural crops in order to determine if this research finds within it are applicable to divergent commodity dynamics and trade circumstances. Conducting such cross-comparison investigations would greatly enhance the external generalizability of this analysis while allowing for more integrated agricultural policymaking.”* |
| **Literature Review**   - **Limitation**: The literature review does not adequately compare the current study's contributions to prior research, nor does it highlight its novelty. - **Suggestion**: Expand the literature review to include a critical assessment of gaps in prior studies and explicitly state how the current research fills these gaps. | We agree with you in this point, and we duly adhere with the suggestions given and truly appreciating. According to we added the following insights to the literature review in the beginning, so the flow of the literature is clear.  **(line 126-140 in the revised manuscript)**  “*Previous studies examining spice export determinants have largely focused on individual factors or specific countries, lacking an integrated, comparative framework. [97] analysed spice exports in Ethiopia using a VECM model, identifying real exchange rates and trade openness as significant long-run determinants. However, his study did not assess domestic consumption or cultivated land area, key supply-side factors. Similarly, [96] found that production volume and domestic consumption significantly affected Indonesia’s pepper exports, but the exchange rate had no significant impact, and cultivated land area was omitted. In the Indian context, [98] provided trend analysis of production and exports but did not empirically assess the drivers of export income. These studies underscore a critical gap: a lack of comprehensive, multi-country analyses linking PV, DC, ER, CLA to CEI. The current study fills this gap by systematically* *comparing the world’s top five cinnamon producers and integrating all four variables into a unified econometric framework, offering novel insights into cross-national differences and actionable policy implications*.” |
| **Language and Formatting**  o Limitation: The manuscript contains instances of awkward phrasing and grammatical errors, which may detract from its professional presentation.  o Suggestion: Perform thorough proofreading and editing to improve clarity and coherence. | Thank you for drawing my attention to this issue. I have carefully examined and edited the manuscript to correct grammatical errors and improve the language to ensure better clarity and consistency throughout the document. |

**Reviewer #2 comments -** **Response Sheet**

| **Reviewer comments** | **Author response to reviewer’s comments** |
| --- | --- |
| You wrote a good article for the improvement of agricultural international trade. Unfortunately, you could not show the novelty of this study well. In addition, you used the SLR method, while secondary data has non-stationary data that cause spurious regression. This issue cannot be solved with SLR but must be solved with advanced econometric models that adopt unit root tests. For that, you need to consider advanced econometric models such as ARDL, ECM or others. | We are sincerely grateful for the reviewer’s encouraging comments and critical observations.  **1. On Novelty Clarification:**  We acknowledge the initial lack of emphasis on the novelty of this research. Accordingly, we have revised the Introduction and Literature Review sections to more clearly state the contribution of this study, specifically:   - This is the first known study to comparatively assess cinnamon export income (CEI) across five top-producing countries, using four macro- and agri-economic variables (PV, DC, CLA, and ER). - Prior research has generally focused on individual countries or single determinants; our study integrates these into a unified framework to offer comparative insights.   **2. On Model Appropriateness & Non-Stationarity Concerns:**  We fully agree with the reviewer that non-stationary time-series data can lead to spurious regression results when using SLR. However, due to limited uniform time-series observations and structural gaps across countries (e.g., missing years), we were constrained from applying advanced models like ARDL or ECM in this iteration.  That said, we have:   - Acknowledged this limitation explicitly in the revised "Limitations and Future Research" section.   **(Line 819-828 in the revised manuscript)**  “*While this study provides valuable insights into the determinants of cinnamon export income across five major producing countries, several methodological limitations must be acknowledged. The use of SLR was chosen for its interpretability and due to constraints in consistent time-series data across countries; however, this approach may not fully capture the complex, potentially nonlinear relationships between variables or account for non-stationarity. As the dataset spans three decades, issues such as spurious regression arising from non-stationary data cannot be ruled out. Future research could address this limitation by employing more advanced econometric techniques such as panel data regression models, nonlinear specifications, or structural equation modeling to better understand dynamic interactions and control for country-specific effects.”*   - Suggested the adoption of ARDL or ECM models as a strong direction for future research, especially when country-specific or pooled datasets become more complete.   **(Line 829-835 in the revised manuscript)**  *“Additionally, models such as the Autoregressive Distributed Lag or Error Correction Model can help account for unit roots and distinguish between short- and long-run relationships. Incorporating interaction terms (e.g., exchange rate × global demand shocks) and testing for multicollinearity using variance inflation factors would also enhance the robustness of future analyses. Addressing these methodological concerns would improve statistical validity and offer deeper insights into the causal mechanisms driving agricultural export performance.”*  We are confident that these clarifications strengthen the manuscript's transparency and rigor and appreciate the reviewer’s guidance. |
