# Supplementary material for: Unlocking cinnamon export success: Key determinants from the world’s top five producers
Source: PLoS One. 2025 Dec 11;20(12):e0338601. doi: 10.1371/journal.pone.0338601 (PMC12697966; doi:10.1371/journal.pone.0338601)
Supplement: Attachment — Submitted filename: Author_Response_Document_auresp_2.docx [file pone.0338601.s004.docx]

**Point-by-Point Response to the Editor and Reviewers**

Dear Academic Editor and Reviewers,

We sincerely thank you for your thoughtful and constructive comments on our manuscript. We greatly appreciate the time and effort you have invested in reviewing our work. Your insights have been instrumental in helping us improve the quality and clarity of the paper. We have carefully considered each comment and have revised the manuscript accordingly. All responses and revisions are detailed point by point in the following document.

All the mentioned line numbers have been addressed and are clearly marked in **the revised manuscript without track changes document.**

**Academic Editor’s comments -** **Response Sheet**

| **Editor comments** | **Author response to Editors’s comments** |
| --- | --- |
| Authors should check the manuscript organization/structure with respect to the journal guidelines (Manuscript organization: Title, Abstract, Introduction, Materials and Methods, Results, Discussion, and Conclusions) and revise the manuscript.  The introduction section is too long, please reduce the general statements and strict to the rationale of the study objectives.  Authors should avoid sub headings in the introduction section and should reduce the introduction section. | We are much appreciated for bringing this in to our attention and we fully agree with your comments given. Accordingly, we have put out maximum effort to avoid subheadings as mentioned, to align our paper with the structure of your journal given. And accordingly, the current paper structure contains the main headings Introduction, Literature Review, Data and Methodology, Results and Discussion, Conclusion and Recommendations.  We hope that now our paper is properly aligned with the guidelines given and the comments. Thank you so much for taking your time to provide this valuable feedback for improving the manuscript. |

**Reviewer #2 comments -** **Response Sheet**

| **Reviewer comments** | **Author response to reviewer’s comments** |
| --- | --- |
| This article is very good but unfortunately you missed the unit root test which is mandatory for secondary data. | Thank you for the comment given on the unit root analysis and hereby authors duly agree with the comment as unit root test is a mandatory requirement when analyzing secondary data. We have mentioned the results of the unit root analysis in the revised manuscript as follows. **(Refer the lines 618-634 in the revised manuscript)** *“Before running the simple linear regression, the stationarity characteristics of the variable were checked through the application of the Levin–Lin–Chu (LLC) test on unit roots. The test was applied on the entire dataset for all countries. The LLC test results are presented in Table 3.* *Table 3. Average values calculated for CEI, DC, PV, CLA and ER* **Variable** \| **1^st^ Difference Variable** \| **Level LLC** \| **First Difference (LLC)**; Log_CEI \| D_CEI \| -0.44 \| -12.03***; Log_DCP \| D_DCP \| -1.59* \| -14.02***; Log_PV \| D_PV \| -1.25 \| -7.84***; Log_CLA \| D_CLA \| 3.07 \| -12.13***; Log_ER \| D_ER \| 0.34 \| -5.75*** *Note: LLC unit root test - H₀: series contains unit roots and H₁: series is stationary. The symbols ***, **, * represent 1%, 5%, and 10% significance levels, respectively.* *Source: Authors’ calculations based on panel data using R ‘plm’ package.* *From the level form analysis, the null hypothesis of non-stationarity could not be rejected for any of the variables namely, Log_CEI, Log_PV, Log_CLA, and Log_ER, at the 5% level of significance, while for Log_DCP, it showed marginal significance at 10% level. This shows that a good portion of the variables exhibit unit root properties in the level form, thus implying non-stationarity. Nevertheless, when the first differencing transformation was applied, all variables D_CEI, D_DCP, D_PV, D_CLA, and D_ER significantly rejected the null hypothesis of a unit root at the 1% significance level. This outcome suggests that the differenced series exhibit stationarity, thereby allowing for the conclusion that all variables can be classified as integrated of order one, I(1).”* |
