# Supplementary material for: Unlocking cinnamon export success: Key determinants from the world’s top five producers
Source: PLoS One. 2025 Dec 11;20(12):e0338601. doi: 10.1371/journal.pone.0338601 (PMC12697966; doi:10.1371/journal.pone.0338601)
Supplement: Attachment — Submitted filename: Author_Response_Document_auresp_3.docx [file pone.0338601.s005.docx]

**Response to the Editor**

Dear Academic Editor,

We sincerely thank you for your thoughtful and constructive comments on our manuscript. We greatly appreciate the time and effort you have invested in reviewing our work. We have carefully considered each comment and have revised the manuscript accordingly. All responses and revisions are detailed point by point in the following document.

All the mentioned line numbers have been addressed and are clearly marked in **the revised manuscript without track changes document.**

**Editor comments -** **Response Sheet**

| **Editor comments** | **Author response to Editor’s comments** |
| --- | --- |
| The revised manuscript is not satisfactory with respect to the manuscript organization. As per the journal guideline, research article manuscript should be organized with the content of abstract, introduction, materials and methods, results and discussion, and conclusions (optional). Please check the manuscript structure and authors should revise the manuscript with respect to the journal guidelines for manuscript organization.  As research article, remove elaborated literature review with the subheading “Literature review” in the introduction section.  Check the subheadings “Data and methodology”, “Results and Discussion” and “Conclusion and recommendations” and write appropriate subheadings and revise the manuscript as per the journal guidelines for manuscript organization.  Please check errors in scientific name of plants (italic, capital & small letter fonts) throughout the manuscript (e.g., Line 116) and revise. | We are grateful to the editorial team that has given us valuable constructive comments. With due consideration to the prescribed format of a scientific article in the journal, we have revised our manuscript thoroughly to cover the following important sections: Abstract, Introduction, Methods, Results & Discussion, and Conclusion & Recommendations.  As mentioned in the comments, we have taken necessary steps to remove the Literature Review with the mentioned subheading from the introduction section. The conceptual framework and the justification included in the methodology section **(Refer the lines 188-196 in the revised manuscript without TC)**  Moreover, greater care has been taken in the refinement of the subheadings:  “Data and Methodology” has been changed to “Methods”,  “Results and Discussion” sections are properly organized in a combined analysis,  “Conclusion and Recommendations” is retained as a concluding section, keeping “Conclusion” optional according to the flexibility of the journal.  This new format not only follows the parameters of the journal submission, but in fact, improves the readability, scientific accuracy, and coherency of the piece as well. This new format will help the readers understand the rationale, methodology, results, and implications in a concise form that we believe is most appropriate to present this multifaceted analysis of export income of cinnamon.  Lastly, the scientific names of all the plants have been corrected to ensure appropriate formatting according to botanical naming conventions (e.g., *Cinnamomum Zeylanicum*).  **(Refer the lines 104 & 116 in the revised manuscript without TC)**  We are confident that these changes will satisfy the journal’s requirements, and we appreciate the continued support. |
