# Supplementary material for: Unlocking cinnamon export success: Key determinants from the world’s top five producers
Source: PLoS One. 2025 Dec 11;20(12):e0338601. doi: 10.1371/journal.pone.0338601 (PMC12697966; doi:10.1371/journal.pone.0338601)
Supplement: Attachment — Submitted filename: Author_Response_Document_auresp_4.docx [file pone.0338601.s006.docx]

**Response to the Editor**

Dear Academic Editor,

We sincerely thank you for your thoughtful and constructive comments on our manuscript. We greatly appreciate the time and effort you have invested in reviewing our work. We have carefully considered each comment and have revised the manuscript accordingly. All responses and revisions are detailed point by point in the following document.

All the mentioned line numbers have been addressed and are clearly marked in **the revised manuscript without track changes document.**

**Editor comments -** **Response Sheet**

| **Editor comments** | **Author response to Editor’s comments** |
| --- | --- |
| Line 123-124: Write expansion of PV, CLA, ER and DC.  Line 129: Write expansion of CEI.  The conclusion section is too elaborative. Should brief the conclusions with respect to the highlight of the obtained results and write recommendations in few lines only. | Thank you for your valuable comments and suggestions. We have carefully revised the manuscript in accordance with your feedback. The full forms of the abbreviations PV, CLA, ER, and DC have now been included in Lines 123–124, and the expansion of CEI has been added in Line 130. Additionally, the conclusion section has been shortened to focus on the key findings of the study, and the recommendations have been rewritten to be more concise. We appreciate your thoughtful review and believe that these changes have improved the clarity and quality of the manuscript. |
